# Retraction: *SAMMSON* drives the self-renewal of liver tumor initiating cells through EZH2-dependent Wnt/β-catenin activation

**DOI:** 10.18632/oncotarget.28307

**Published:** 2022-11-02

**Authors:** Xiaopeng Li, Ming Li, JiaLi Chen, Hua Dai, Liang Wang, Ying Xiong, Yuanbin Zhong, Lunli Zhang

**Affiliations:** ^1^Department of Infectious Disease, The First Affiliated Hospital of Nanchang University, Nanchang, JiangXi 330006, China; ^2^Department of Function, JiangXi Children’s Hospital, Nanchang, JiangXi 330006, China; ^3^Department of Pathology, The First Affiliated Hospital of Nanchang University, Nanchang, JiangXi 330006, China; ^*^These authors have contributed equally to this work

**This article has been retracted:** Figure 6B contains an image previously published in an earlier paper from another journal. The authors have been unable to retrieve their original experimental data, nor can they produce the necessary ethics approval documentation. Based on these deficiencies, all authors have agreed to retract this paper from Oncotarget.

Original article: Oncotarget. 2017; 8:103785–103796. 103785-103796. https://doi.org/10.18632/oncotarget.21792

